# The Synergistic Effects of Fine Particulate Matter and High Humidity on Allergic Asthma: An Association with TRPV4/MAPK Pathway Activation

**DOI:** 10.3390/toxics14030219

**Published:** 2026-03-03

**Authors:** Ziyu Shu, Xu Yang, Baizhan Li, Ping Ma, Yang Wu, Yan Li, Miao Guo, Chenqiu Du, Fangxin Fang, Runming Yao

**Affiliations:** 1 Joint International Research Laboratory of Green Buildings and Built Environments (Ministry of Education), Chongqing University, Chongqing 400045, China; ziyushu@outlook.com (Z.S.); baizhanli@cqu.edu.cn (B.L.); guomiao@tcu.edu.cn (M.G.); duchenqiu90@163.com (C.D.); 2 Hubei Key Laboratory of Environmental Risks and Related Diseases Precision Control, Hubei University of Science and Technology, Xianning 437100, China; yangxu@mail.ccnu.edu.cn (X.Y.); wysj2007@126.com (Y.W.); ly18281300536@163.com (Y.L.); 3 Institute of Eastern-Himalaya Biodiversity Research, Dali University, Dali 671003, China; 4 Department of Pharmacy, Ezhou Central Hospital, Ezhou 436000, China; 5 School of Energy and Safety Engineering, Tianjin Chengjian University, Tianjin 300384, China; 6 Department of Earth Science and Engineering, Imperial College London, London SW7 2AZ, UK; f.fang@imperial.ac.uk; 7 School of the Built Environment, University of Reading, Reading RG6 6DB, UK

**Keywords:** allergic asthma, fine particulate matter, high relative humidity, TRPV4 pathway, MAPK (p38 MAPK, JNK, ERK1/2) pathway

## Abstract

Identifying environmental factors contributing to allergic asthma is critical for effective prevention. PM_2.5_, a major environmental pollutant, and high relative humidity frequently coexist in urban and industrialized regions, particularly when ventilation is poor. However, the combined effects of PM_2.5_ and humidity remain unclear. This study used a murine asthma model, exposing male Balb/c mice sensitized with ovalbumin (OVA) to PM_2.5_ (75 μg/m^3^ and 35 μg/m^3^), based on China’s Ambient Air Quality Standards (GB3095-2012), and/or varying relative humidity levels in a controlled chamber. Allergic asthma severity was evaluated through histopathological changes, pulmonary function, Th1/Th2 balance, mucus hypersecretion, and inflammatory factor levels, alongside the activation of TRPV4 and MAPK signaling pathways (ERK, p38MAPK, and JNK). The results showed that high humidity (90%) or PM_2.5_ exposure alone had minimal impact, but combined exposure to 75 μg/m^3^ PM_2.5_ and 90% humidity markedly aggravated airway hyperresponsiveness, inflammation, and mucus hypersecretion. These changes coincided with enhanced TRPV4 activation and MAPK signaling, particularly p38MAPK and JNK, while ERK1/2 remained unaffected. A lower PM_2.5_ concentration (35 μg/m^3^) combined with 90% humidity had a weaker impact. Blocking TRPV4 with HC-067047 effectively mitigated asthma exacerbation caused by combined exposure. These findings demonstrate that co-exposure to PM_2.5_ and high humidity dose-dependently exacerbates allergic asthma, an effect likely mediated by TRPV4-MAPK pathway activation. Targeting TRPV4 may offer a potential therapeutic strategy to mitigate asthma exacerbation in environments with high humidity and PM_2.5_.

## 1. Introduction

As the global climate changes, exposure to multiple atmospheric pollutants and environmental triggers may have synergistic health effects [1,2]. As a fundamental climatic factor, relative humidity (RH) is rarely the explicit focus in health impact studies [3], yet asthma affects 262 million people globally, causing 455,000 deaths annually [4]. High humidity can directly trigger airway-narrowing reflexes, alter mucus viscosity, and modulate airborne pollutant behavior [5], with indoor RH above 60% associated with increased respiratory symptoms [6].

Airborne fine particulate matter (PM_2.5_), formally defined as a carcinogen by WHO/IARC [7], originates from diverse outdoor (traffic emissions, solid-fuel burning) and indoor sources (cooking, tobacco smoke), with significant infiltration of outdoor particles into residential environments [8]. These particles bypassed mucosal barriers to penetrate deep into lungs, inducing chronic inflammation and oxidative stress that promoting Th2-skewed immune response [9,10,11]. Exposure to elevated PM2.5 and humidity is prevalent in urban areas with poor ventilation, where PM2.5 frequently exceeds standards and RH levels can reach 90% in subtropical settings [12,13], making elucidation of their synergistic molecular mechanisms essential.

Direct humidity sensation in mammals remains poorly understood [14]. We hypothesize that it is plausible that environmental humidity indirectly activates Transient Receptor Potential Vanilloid 4 (TRPV4) through alterations in epithelial microenvironment osmolarity. TRPV4 is a key ion channel and a sensory pioneer capable of responding to osmotic pressure, temperature, and mechanical stimuli [15,16]. In the respiratory system, TRPV4 is highly expressed in airway epithelial cells and alveolar types I and II, where it maintains barrier integrity, mediates calcium influx, and regulates ciliary activity [17,18]. High environmental humidity may influence periciliary fluid osmolarity and the mechanical properties of the epithelial surface, thereby indirectly modulating TRPV4 activation [19]. Activation of TRPV4 modulates calcium flux, influencing airway remodeling, goblet cell metaplasia, and fibrotic progression [20]. Consequently, we hypothesize that TRPV4-dependent pathways mediate the aggravated asthmatic responses provoked by PM2.5 and high humidity. Calcium influx triggered by TRPV4 activates downstream Mitogen-Activated Protein Kinases (MAPKs), including ERK, p38, and JNK [21]. While ERK and p38 are linked to smooth muscle proliferation and cytokine production (e.g., TNF-α, IL-8), JNK activation influences immune cell activation and airway hyperresponsiveness [22,23]. Although p38 MAPK is a known driver of asthma exacerbated by ozone, the specific connections between TRPV4 and the ERK/JNK pathways under combined PM2.5 and humidity stress remain unclear.

This study addresses three research questions: (1) Do single or combined exposures contribute to asthma exacerbation? (2) Which biomarkers indicate co-exposure health impacts? (3) What molecular mechanisms underlie PM_2.5_–high humidity synergy? Using an ovalbumin (OVA)-induced murine model, we evaluated pulmonary function, airway remodeling, and TRPV4-MAPK activation, utilizing the specific inhibitor HC-067047 to assess therapeutic potential.

## 2. Materials and Methods

### 2.1. Exposure to PM_2.5_ and High Humidity

PM_2.5_ was suspended in sterile saline with 0.05% Tween-80 and ultrasonically dispersed before intranasal administration to mice. Based on China’s “Ambient Air Quality Standards” (GB3095–2012) [24], the 24 h PM_2.5_ concentration limits are 35 μg/m^3^ (class 1) and 75 μg/m^3^ (class 2). To translate environmental exposure into biologically relevant murine doses, we employed the FDA-recommended allometric scaling method based on Body Surface Area (BSA) [25]. Based on an individual’s tidal volume of 12 m^3^/day, the daily PM_2.5_ intake was estimated as 75 μg/m^3^ × 12 m^3^/day = 900 μg/day. The human-equivalent dose (HED) was converted to mouse dose using the formula Mouse dose (mg/kg) = HED (mg/kg) × (Human Km/Mouse Km), where Km factors are 37 for humans (60 kg) and 3 for mice (0.02 kg), yielding a conversion factor of 12.3. We accounted for fractional deposition of fine particles in the deep lung (estimated at 10% for PM2.5 < 1.8 μm) [26], calculating effective pulmonary deposition as 900 μg/day ÷ 60 kg = 15 μg/kg/day for humans and an adjusted mouse dose = 15 μg/kg × 12.3 × 0.10 ≈ 18 μg/kg/day. Given the once-daily intranasal administration in our protocol, the final doses of 0.07 mg/kg/day and 0.15 mg/kg/day were selected to bracket physiologically relevant exposures at approximately 4–8 times the calculated effective dose, consistent with accelerated toxicological testing protocols that compress chronic environmental exposures into acute experimental timeframes) [25]. Arizona A2 test dust (ISO 12103–1 standard) comprising 90% particles smaller than 1.752 µm served as a respirable PM_2.5_ surrogate [27]. The composition included inorganic ions and mineral elements, confirmed by the supplier’s certificate of analysis (CoA), as summarized in Table 1. Timewise, PM_2.5_ was administered intranasally between 08:00 and 09:00 to simulate typical real-world exposure during office hours.

For humidity settings, peak indoor humidity reached 90% in seven of nine surveyed Chinese cities [28], while optimal respiratory humidity ranges from 40 to 60% [29]. To investigate humidity effects, mice were exposed to either 50% (control) or 90% (high) relative humidity for 12 h per day. Two artificial climate chambers (PRX800C, Shanghai, China) maintained these environments at 25 ± 1 °C and a ventilation rate of 3 L/min, monitored by HOBO sensors, ensuring relative humidity levels of either 45 ± 5% or 90 ± 5%.

### 2.2. Animal Exposure Protocols

Five-week-old male Balb/c mice (SPF grade, 16–19 g BW) were obtained from the Hubei Province Experimental Animal Center (certificate No. 42000600029218). They were housed under standard conditions, including a 12 h light–dark cycle, 50–70% humidity, and a temperature range of 20–25 °C, with unrestricted access to food and water (approval ID: HBUST-IACUC-2022-12-080).

**Figure 1:**
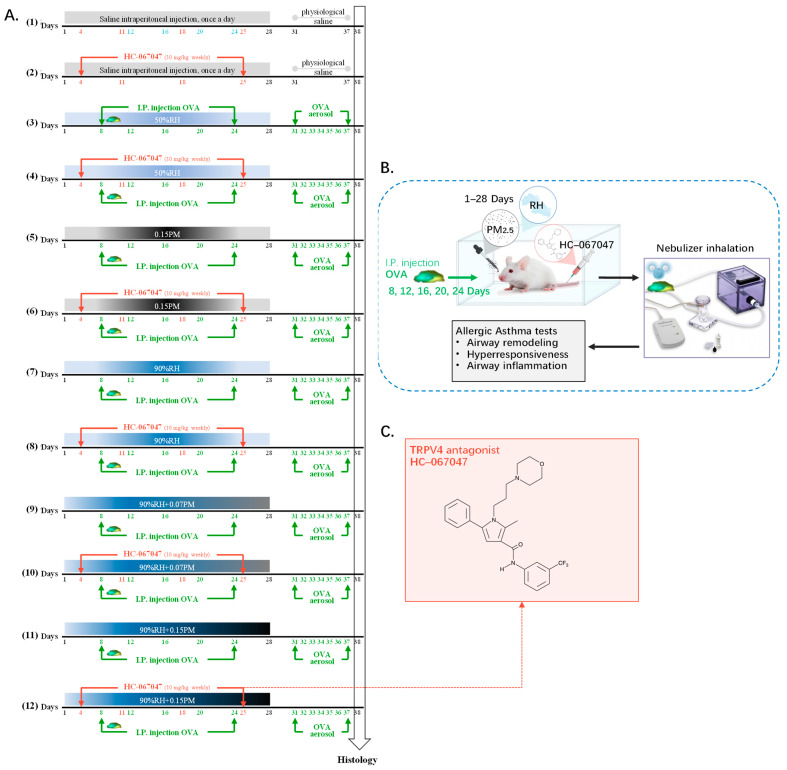
The experimental protocol. (**A**) Schematic timeline of sensitisation, challenge and exposure for the 12 experimental groups; (**B**) Schematic overview of the in vivo exposure system; (**C**) Chemical structure of the TRPV4 antagonist HC-067047.

A total of 144 mice were randomly divided into 12 experimental groups (Figure 1). The allergic asthma model was generated using ovalbumin (OVA) sensitization. Mice received intraperitoneal (i.p.) injections of 5 mg OVA mixed with 175 mg Al(OH)_3_ in 30 mL saline on days 8, 12, 16, 20, and 24. To provoke asthma symptoms, they were exposed to a 1% OVA aerosol for 30 min daily during the final week. Mice in the HC-067047 treatment groups received weekly intraperitoneal injections (i.p.) at 10 mg/kg body weight (bw) [30], administered post exposure to PM_2.5_ under controlled humidity conditions. On day 38, all mice were euthanized, and lung tissues, serum, and bronchoalveolar lavage fluid (BALF) were harvested for biomarker detection and histopathological examination.

(1) Saline exposure group and normal 50% relative humidity (saline group). 

(2) HC-067047 blocked group (saline + HC group).

(3) OVA modeling group (OVA group). 

(4) OVA modeling and blocked group (OVA + HC group). 

(5) OVA modeling and high-concentration PM exposure group (OVA + 0.15PM). 

(6) OVA modeling and high-concentration PM exposure and blocked group (OVA + 0.15PM+ HC group). 

(7) OVA modeling and high relative humidity exposure group (OVA + 90%RH group). 

(8) OVA modeling and high relative humidity exposure and blocked group (OVA + 90%RH + HC group). 

(9) OVA modeling group exposed to low-concentration PM and high relative humidity (OVA + 0.07PM group + 90%RH). 

(10) OVA modeling and blocked group exposed to low-concentration PM and high relative humidity, treated with HC-067047 (OVA + 0.07PM + 90%RH +HC group). 

(11) OVA modeling group exposed to high-concentration PM and high relative humidity (OVA + 0.15PM group + 90%RH). 

(12) OVA modeling and blocked group exposed to high-concentration PM and high relative humidity, treated with HC-067047 (OVA + 0.15PM + 90%RH + HC group).

### 2.3. Serum Isolation and Tissue Preparation

After a 37-day experimental period, 7 mice per group were humanely euthanized via lethal-dose intraperitoneal injection of sodium pentobarbital (100 mg/kg bw). Following thoracic exposure, the chest wall was disinfected with ethanol-saturated cotton prior to cardiac puncture. Nlood (0.8–1.0 mL) was collected via cardiac puncture using sterile syringes, transferred to 1.5 mL tubes, and centrifuged (3000 rpm, 10 min, 25 °C). The serum aliquots were stored at −80 °C for subsequent quantification of total immunoglobulins E (IgE), OVA-specific IgE, OVA-specific IgG1, interleukin (IL)-4 levels (Th2-mediated immune hyperactivation), and Interferon-γ (IFN-γ) (Th1-associated immune dysregulation) [31]. Subsequently, lungs were excised post mortem, perfused with cold 1× phosphate-buffered saline (PBS; pH 7.4), and blotted dry. The right lung was homogenized in PBS (10% *w*/*v*), whereas the left lung underwent centrifugation (10,000 rpm, 10 min, 4 °C), with supernatants archived at −80 °C. This lung supernatant was utilized to determine levels of various signaling molecules and transcription factors involved in macrophage activation and innate immune regulation. Specifically, we assessed levels of Transient Receptor Potential Vanilloid 4 (TRPV4), along with components of the Mitogen-Activated Protein Kinase (MAPK) cascade: p38 Mitogen-Activated Protein Kinases (p38 MAPKs), c-Jun N-terminal Kinase (JNK), and Extracellular Signal-Regulated Kinase 1/2 (Erk-1/2). Additionally, transcription factors essential for immune modulation and oxidative stress regulation, namely Activator Protein 1 (AP-1), Nuclear Factor kappa-light-chain-enhancer of activated B cells (NF-κB), and Nuclear Factor (Erythroid-derived 2)-like 2 (Nrf2), were evaluated.

### 2.4. Pathology and Immunohistochemistry of Lung Sections

The right lung was preserved in 4% paraformaldehyde (pH = 7.4) solution for subsequent histological evaluation. The tissue underwent sequential immersion in graded ethanol and xylene before being embedded in paraffin, adhering to the standard laboratory protocol. After 24 h, 4 μm thick sections were prepared, deparaffinized, and mounted on glass slides. Hematoxylin and eosin (H&E) staining was performed to examine general morphology, followed by immunohistochemistry for TRPV4 expression. For immunohistochemical processing, sections were rehydrated after being deparaffinized using xylene and graded ethanol, and antigen retrieval was conducted with a sodium citrate buffer. To minimize non-specific binding, slides were incubated with 3% bovine serum albumin (BSA) in PBS containing 0.05% (*v*/*v*) Tween-20 (PBST) at room temperature for 1 h, followed by overnight exposure to rabbit anti-TRPV4 polyclonal antibody (Alomone Labs, Jerusalem, Israel; 1:100) at 4 °C. The next day, biotinylated goat-anti-rabbit HRP secondary antibody (1:200) was applied at room temperature for 1 h, followed by hydrogen peroxide treatment and diaminobenzidine (DAB) staining. Counterstaining with hematoxylin was performed before air-dying the slides, which were subsequently covered with a water-soluble mounting medium for further microscopic analysis [32].

### 2.5. Pulmonary Function Tests and BALF Collection

At 24 h after the final OVA aerosol challenge on day 38, 5 mice per group were tested for pulmonary function. The animals were anesthetized with intraperitoneal injection of sodium pentobarbital (100 mg/kg). A vertical incision was made in the neck to access the trachea, where a catheter was placed and linked to a mechanical ventilator. Ventilator settings were adjusted to maintain a 1.5:1 respiratory-to-inspiratory ratio and a breathing rate of 90 cycles per minute. To assess airway hyperresponsiveness (AHR), methacholine (MCH, 50 μL) was administered intravenously via the jugular vein at progressively increasing concentrations of 0.025, 0.05, 0.1, and 0.2 mg/kg at 5 min intervals. Respiratory mechanic parameters, namely inspiratory resistance (Ri), expiratory resistance (Re), and lung dynamic compliance (Cldyn), were recorded in real time using the AniRes 2005 lung function system (Bestlab, version 2.0, China). The R area was calculated as the area under the curve between peak Ri or Re and the baseline measurements during the 300 s interval following methacholine challenge. Cldyn was quantified using the minimum recorded value during forced oscillatory maneuvers [30]. Following the pulmonary function tests, bronchoalveolar lavage fluid (BALF) was collected. The lungs were lavaged three times with 1 mL of cold phosphate-buffered saline (PBS). The recovered fluid was pooled then centrifuged (1000 rpm, 10 min, 4 °C). The supernatant was transferred into new microfuge tubes and stored at −80 °C for cytokine analysis. The remaining cell pellets were resuspended in PBS, and the cells were stained using Wright–Giemsa stain to enhance visualization. Eosinophil counts were conducted using an oil immersion light microscope at 100× magnification. To ensure the reliability of the results, two independent, blinded observers performed cell quantification. The counts recorded by both observers were averaged to minimize variability and enhance accuracy [33].

### 2.6. Main Reagents and Kits

The following reagents were used: Ovalbumin (OVA), methacholine (MCH), sodium pentobarbital, and paraformaldehyde solutions were obtained from Sigma-Aldrich (St. Louis, MO, USA). Tween-80 (CAS: 9005–65-6) was obtained from Amresco (Solon, OH, USA). Arizona test dust (A2) was supplied by Beckman Coulter Inc. (Shanghai, China). HC-067047 was sourced from Yuanye Bio-Technology Co. Ltd. (Shanghai, China). Disposable Cell Counting Plates were obtained from Watson Co., Ltd. (Tokyo, Japan), and Giemsa stain solution was procured from Thermo Fisher Scientific Inc. (Waltham, MA, USA). ELISA kits for TRPV4, p38 MAPK, JNK, Erk-1/2, AP-1, NF-κB, and Nrf2 were supplied by Enzyme-linked Biotechnology Co., Ltd. (Shanghai, China). ELISA kits for assessing key markers of allergic asthma, including T-IgE, OVA-IgE, OVA-IgG1, IL-4, IL-5, IL-13, IL-25, and IL-33, were purchased from FANKEL Industrial Co., Ltd. (Shanghai, China). Mouse-anti-TRPV4 antibody (catalog number: ab259361) was acquired from Abcam (Cambridge, MA, USA). Spectrophotometric (Power waveXS) and fluorometric (ELx800) microplate analyses were conducted using instrumentation from Bio-Tek (Winooski, VT, USA). A temperature-controlled circulating water bath (HH-42; Changyuan, Beijing, China) and a refrigerated centrifuge (Eppendorf 5424 R; Hamburg, Germany) were employed for thermal regulation and sample processing. Histological imaging utilized an Olympus DP73 brightfield microscope (Tokyo, Japan). All immunoglobulins, cytokines, and signaling proteins were quantified by ELISA using the kits listed above, according to the manufacturers’ instructions.

### 2.7. Statistical Analysis

To minimize observer bias, all morphometric and quantitative analyses were performed by investigators blinded to the experimental group assignments. All statistical analyses were conducted using GraphPad Prism 10.1.2 (San Diego, CA, USA). Data were expressed as the mean ± standard deviation (SD). The distribution of each variable was checked using the Shapiro–Wilk normality test, and homogeneity of variances was assessed via the Brown–Forsythe test. For data that met the assumptions of normality and equal variance, group differences were assessed using one-way analysis of variance (ANOVA) followed by Tukey’s post hoc test for pairwise comparisons. For data that did not satisfy these assumptions, group differences were analyzed using the Kruskal–Wallis test followed by Dunn’s post hoc test. For AHR assessment, two-way ANOVA with multiple comparison tests was applied. Statistical significance was defined as *p* < 0.05, while *p* < 0.01 indicated a highly significant difference.

## 3. Results

### 3.1. PM_2.5_ and High Relative Humidity Aggravate Allergic Asthma Symptoms

**Figure 2:**
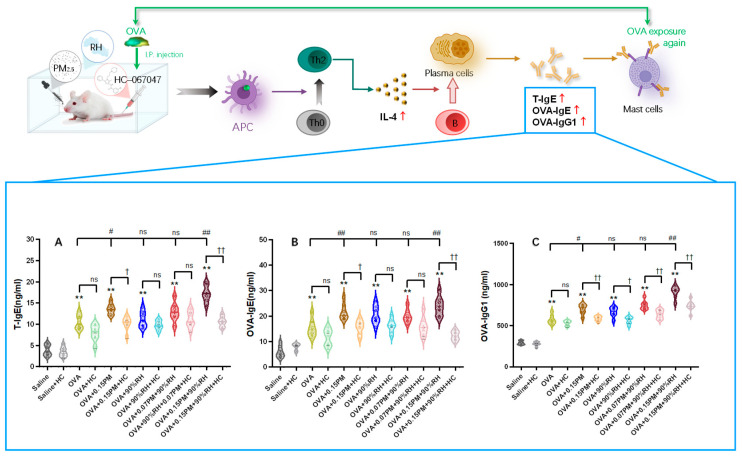
Serum total IgE (T-IgE) (**A**), OVA-specific IgE (**B**), and IgG1 (**C**) levels in the 12 experimental groups. ** *p* < 0.01: a significant difference compared with the saline group. ^ns^ *p* > 0.05, # *p* < 0.05, ## *p* < 0.01: different exposure groups compared with the OVA group. † *p* < 0.05, †† *p* < 0.01: the blocking groups compared with the corresponding exposure groups (*n* = 7).

Figure 2 illustrates the effects of various experimental conditions on serum levels of serum total IgE (T-IgE), OVA-specific IgE (OVA-IgE), and OVA-specific IgG1 (OVA-IgG1), representing overall allergic status, antigen-specific immediate hypersensitivity (allergic response), and chronic sensitization with adaptive immunity, respectively.

In comparison to the saline group, both OVA modeling groups show a significant elevation in T-IgE and OVA-IgE levels (*p* < 0.01), confirming the successful induction of an allergic response. The OVA + 0.15PM group exhibits significant increases in these markers compared to the OVA-only group. When PM_2.5_ and high humidity were combined (e.g., OVA + 0.15PM + 90%RH), the levels of T-IgE, OVA-IgE, and OVA-IgG1were the highest among all groups, consistent with a synergistic interaction between these environmental factors. The low-concentration PM and high humidity (OVA + 0.07PM + 90%RH) exposure group shows a comparatively smaller increase compared to the high-concentration PM (OVA + 0.15PM + 90%RH) group, highlighting that high-concentration PM significantly amplifies the allergic response to OVA, while low-concentration PM does not exhibit such a strong effect. HC-067047 effectively mitigates allergic reactions to some extent, especially with the combination of environmental factors, producing *p* < 0.01.

### 3.2. Influences of PM_2.5_ and High Relative Humidity on AHR

**Figure 3:**
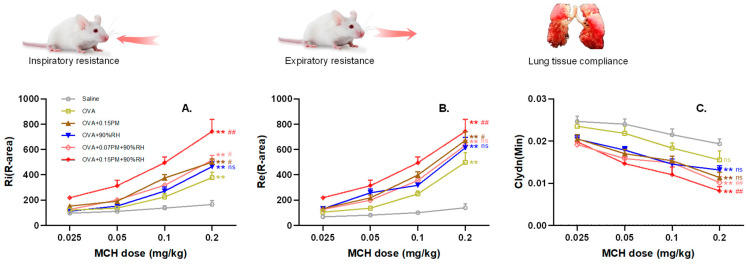
Pathological injury and AHR changes after exposure to different levels of PM_2.5_ and/or different relative humidities. (**A**–**C**) Ri, Re, and Cldyn values of the different treatment groups, respectively. ** *p* < 0.01: a significant difference compared with the saline group. ^ns^ *p* > 0.05, ^#^ *p* < 0.05, ^##^ *p* < 0.01: different exposure groups compared with the OVA group. (*n* = 5).

Lung function changes in mice were primarily reflected in alterations in airway resistance during inspiration and expiration, as well as lung compliance (Figure 3). With escalating MCH concentrations, Ri and Re progressively increased and Cldyn decreased. Compared to the saline group, all OVA-sensitized groups showed significant differences (*p* < 0.01), confirming the successful establishment of the allergic asthma model.

Exposure to the group with only PM_2.5_ exacerbated pulmonary dysfunction, Ri and Re were significantly elevated (*p* < 0.01), and Cldyn showed a downward trend compared to the OVA group. However, co-exposure with high humidity (90%RH) was associated with further amplification of these effects. Notably, the high-dose PM_2.5_ group (OVA + 0.15PM + 90%RH) showed the most severe effects, with marked increases in Ri and Re (*p* < 0.01) and a significant reduction in Cldyn (*p* < 0.01). In contrast, the low-dose co-exposure group (OVA + 0.07PM + 90%RH) showed no significant changes (*p* > 0.05) relative to exposure to the PM_2.5_-only group.

### 3.3. Histological Analysis Reveals Lung Changes and Eosinophil Dynamics in BALF

**Figure 4:**
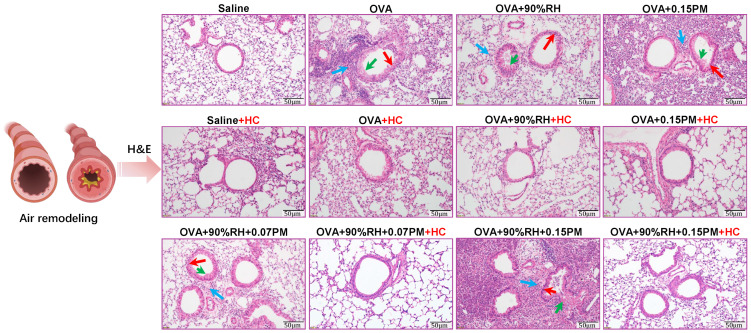
Effect of relative humidity and/or PM_2.5_ on allergic airway inflammation in lung tissues (H&E staining). Arrows illustrate different cells. Green: mucus. Red: goblet cells. Blue: peribronchial inflammation.

Figure 4 illustrates significant histological differences among groups. In comparison to the saline group, the OVA-sensitized group exhibited pronounced inflammatory cell infiltration around bronchial and alveolar regions, extending into bronchial luminal spaces. In the single-exposure groups, the high humidity exposure single-exposure group (OVA + 90%RH) showed epithelial cells and inflammatory secretions shedding into the bronchial cavities. Further, the PM_2.5_-only group (OVA + 0.15PM) showed more severe changes, including wrinkling of the airway walls and greater peribronchial inflammation. However, there was a visible exacerbation of airway remodeling in the co-exposure group (OVA + 0.15PM + 90%RH). In addition, the low-dose PM_2.5_ co-exposure group (OVA + 0.07PM + 90%RH) showed a moderate change. Treatment with HC-067047 led to a modest reduction in inflammatory cell infiltration and airway fibrosis in the OVA + HC group compared to the OVA group, while significant improvements were observed in both the single/combined exposure groups compared to their untreated counterparts.

Lung injury and allergic asthma aggravation is reflected by an increase in the number of eosinophil cells. Eosinophils are primary cellular markers for validating asthma modeling. Table 2 shows that the total cell count and eosinophil count remain minimal in both the saline and saline + HC groups, yet are markedly increased in the PM_2.5_ exposure groups. This accumulation is most pronounced under combined exposure conditions, which supports the histopathological observations previously discussed. Moreover, HC-067047 treatment effectively reduces eosinophil counts across all exposure groups, underlining its therapeutic potential against allergic asthma influenced by environmental factors.

### 3.4. Co-Exposure Modulates Th1 and Th2-Dominant Immunity Responses

**Figure 5:**
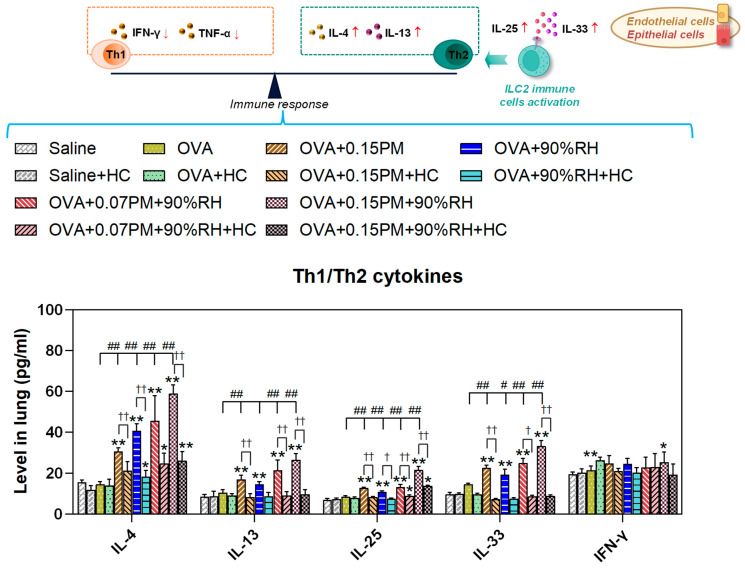
The effects of co-exposure on cytokine levels in lung tissue. Levels of Th2 cytokines (IL-4, IL-13, IL-25, and IL-33) and Th1 cytokines (IFN-γ) across the different treatment groups. * *p* < 0.05, ** *p* < 0.01: a significant difference compared with the saline group. ^#^ *p* < 0.05, ^##^ *p* < 0.01: different exposure groups compared with the OVA group. ^†^ *p* < 0.05, ^††^ *p* < 0.01: the blocking groups compared with the corresponding exposure groups (*n* = 7).

To evaluate the Th1/Th2 balance, Th2 cytokines (IL-4, IL-13, IL-25, IL-33) and the Th1 cytokine (IFN-γ) were quantified in lung tissues under various exposure conditions (Figure 5). The OVA group showed a significant increase in IL-4 compared to the saline group (*p* < 0.01), indicating a shift towards a Th2 response, while IFN-γ levels remained unchanged. Exposure to 0.15PM and 90%RH further elevated Th2 cytokines (*p* < 0.01), especially IL-4 and IL-33, while IFN-γ remained low, maintaining a Th2-skewed response. In the HC-067047-treated groups, IL-4 levels decreased and IFN-γ/IL-4 ratios increased significantly (*p* < 0.01), indicating that TRPV4 inhibition partially restored the Th1/Th2 balance under PM and 90%RH exposure.

### 3.5. Co-Exposure Enhances TRPV4 Activation and Modulates MAPK Pathway Signaling

**Figure 6:**
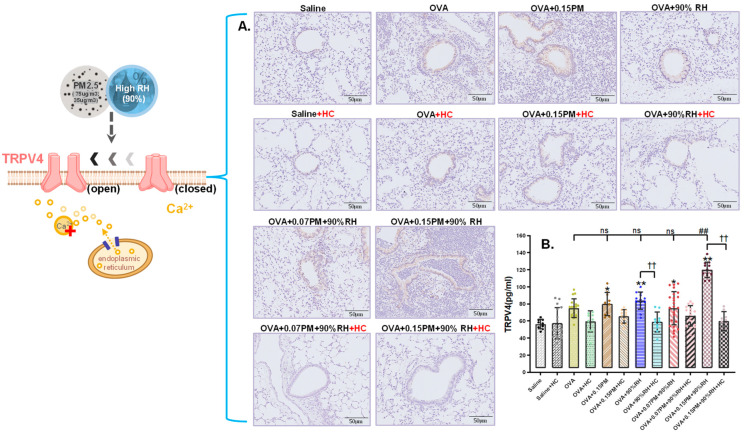
The impact of exposure to PM_2.5_ and different relative humidities on the TRPV4 ion channel in the lung tissue. (**A**) Immunohistochemistry for TRPV4 in the lung tissue (original magnification, 200×). (**B**) The average optical density of TRPV4 in the lung tissue. * *p* < 0.05, ** *p* < 0.01: a significant difference compared with the saline group. ^ns^ *p* > 0.05, ## *p* < 0.01: different exposure groups compared with the OVA group. †† *p* < 0.01: the blocking groups compared with the corresponding exposure groups (*n* = 7).

To assess the involvement of TRPV4 in the exacerbation of allergic asthma induced by PM_2.5_ and high relative humidity, TRPV4 levels were measured in lung tissue using immunofluorescence and ELISA (Figure 6). A significant increase in TRPV4 expression was observed in the OVA group compared to the saline group (*p* < 0.05). Co-exposure to PM_2.5_ and 90%RH was associated with a highly significant increase in TRPV4 expression compared to the OVA group (*p* < 0.01). However, low-concentration PM_2.5_ combined with 90%RH did not cause notable changes in TRPV4 expression, while high-concentration PM_2.5_ combined with 90% relative humidity led to a significant increase in TRPV4 levels. Additionally, the application of TRPV4-specific inhibitors, including HC-067047, resulted in a decrease in TRPV4 and its associated downstream signaling markers across all TRPV4-blocking groups to varying extents.

**Figure 7:**
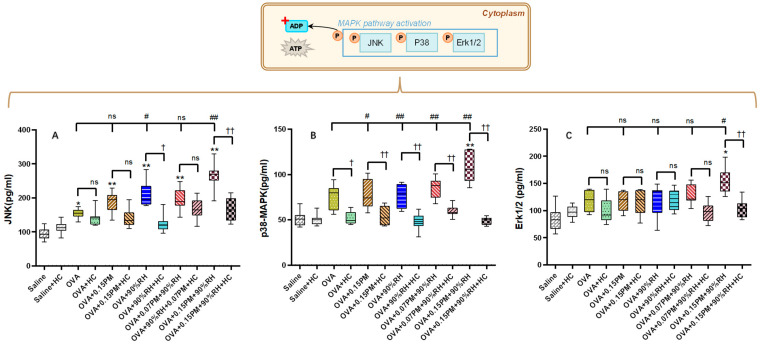
The impact of exposure to PM_2.5_ and different relative humidities on MAPK. (**A**) The average optical density of JNK in the lung tissue. (**B**) The average optical density of phospho-p38 MAPK in the lung tissue. (**C**) The average optical density of ERK1/2 in the lung tissue. * *p* < 0.05, ** *p* < 0.01: a significant difference compared with the saline group. ^ns^ *p* > 0.05, ^#^ *p* < 0.05, ^##^ *p* < 0.01: different exposure groups compared with the OVA group. ^†^ *p* < 0.05, ^††^ *p* < 0.01: the blocking groups compared with the corresponding exposure groups (*n* = 7).

TRPV4 channel activation is associated with an elevated calcium ion level and concurrent activation of downstream signaling cascades. Our study mechanistically interrogates the MAPK pathway (Figure 7), which is a key regulator of inflammation and airway remodeling.

In Panel A, the OVA group exhibited a significant increase in JNK levels compared to the saline group (*p* < 0.01), indicating activation of the stress-related pathway. Exposure to 0.15PM or 90%RH alone further elevated JNK levels (*p* < 0.01), with the combined exposure of 0.15PM + 90%RH resulting in the highest JNK activation. Interestingly, low-concentration PM_2.5_ (0.07PM) combined with 90%RH had no marked effect on JNK levels, while high-concentration PM_2.5_ with 90%RH significantly increased JNK activation beyond that of the OVA-only group (*p* < 0.01). Treatment with HC-067047 significantly reduced JNK levels across all groups (*p* < 0.01), indicating that TRPV4 blockade attenuates this signaling pathway. Panel B shows a similar trend for p38 MAPK activation. The OVA group displayed a marked increase in p38 MAPK levels (*p* < 0.01), which were further elevated in the combined 0.15PM + 90%RH exposure group (*p* < 0.01). Once again, HC-067047 treatment reduced p38 MAPK levels significantly (*p* < 0.01), suggesting a dampened response in the presence of the inhibitor. Panel C shows the ERK1/2 levels, which were less affected by the exposure conditions compared to JNK and p38 MAPK. There were no significant changes in ERK1/2 levels across most groups, except for a slight but significant increase in the 0.15PM + 90%RH + HC group (*p* < 0.05), indicating minimal involvement of ERK1/2 in the response to these environmental stressors.

### 3.6. Environmental Stressors Augment Oxidative Stress Pathways

**Figure 8:**
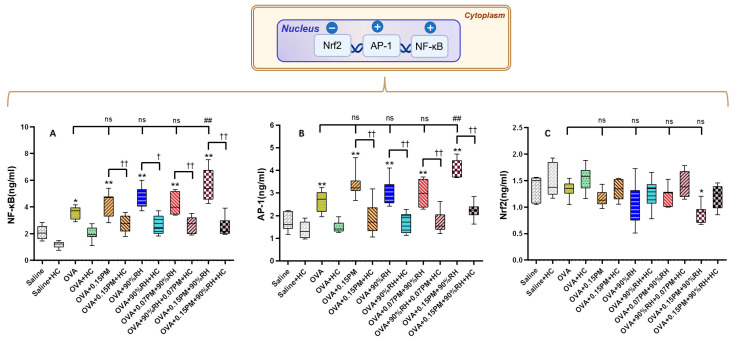
The impact of exposure to PM_2.5_ matters and different relative humidities on inflammation in the allergic tissue. (**A**) The levels of NF-κB. (**B**) The level of AP-1 in the blood tissue. (**C**) The level of Nrf2 in the blood tissue. * *p* < 0.05, ** *p* < 0.01: a significant difference compared with the saline group. ^ns^ *p* > 0.05, ^##^ *p* < 0.01: different exposure groups compared with the OVA group. ^†^ *p* < 0.05, ^††^ *p* < 0.01: the blocking groups compared with the corresponding exposure groups (*n* = 7).

To investigate the connection between oxidative stress and cellular signaling pathways, we measured the levels of AP-1, NF-κB, and Nrf2 in lung tissue across various exposure groups, as shown in Figure 8.

AP-1 and NF-κB levels were significantly higher in the OVA group relative to the saline group (*p* < 0.05), indicating increased inflammation. Exposure to 0.15PM and 90%RH further elevated these markers, suggesting enhanced oxidative stress. HC-067047 treatment reduced AP-1 and NF-κB across all groups (*p* < 0.01), showing its role in limiting TRPV4-driven inflammation. In contrast, Nrf2 levels were lower in the OVA group, with the lowest levels in the 0.15PM + 90%RH group (*p* < 0.01).

## 4. Discussion

**Figure 9:**
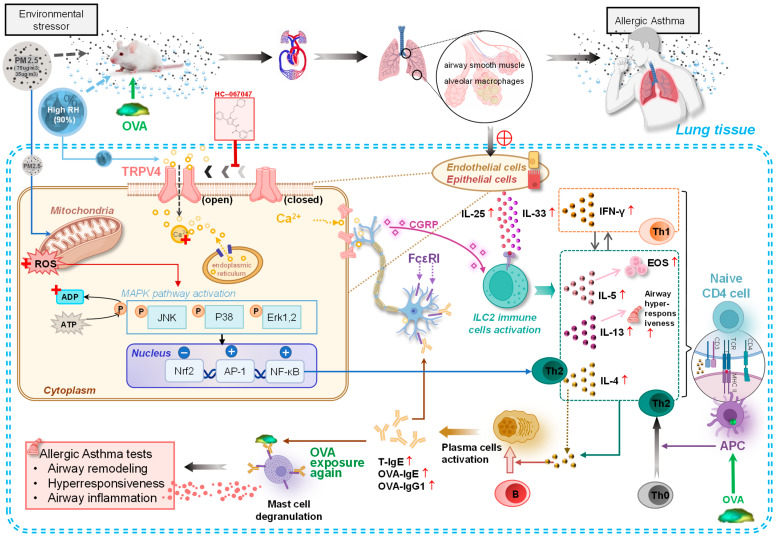
Molecular mechanisms of PM_2.5_ exacerbated asthma in high relative humidity environments.

In this study, we utilized an OVA-induced allergic asthma model to investigate the synergistic effects of PM2.5 and high relative humidity (90% RH). The results revealed the key molecular mechanism of environmental asthma exacerbation through the TRPV4 activation → MAPK phosphorylation → NF-κB inflammatory response pathway, as illustrated in Figure 9. Specifically, we found that high humidity acts as a force multiplier for PM2.5 toxicity, shifting the immune profile toward a Th2-dominant state and promoting severe airway remodeling.

### 4.1. Phenotypic Validation and Immunological Shift

Asthma is characterized clinically by airway wall thickening, inflammatory cell infiltration, and increased mucus secretion [10,34]. Consistent with these features, our histopathological analysis showed that whilst single exposure to PM_2.5_ or 90% RH elicited only mild changes, co-exposure produced marked airway wall remodeling and inflammatory cell accumulation (Figure 4; Table 2). These structural alterations were accompanied by functional impairment, reflected in enhanced airway hyperresponsiveness (AHR), which accords with prior reports linking PM_2.5_ exposure to reduced FEV1/FVC [9], increased airway resistance, and worsening asthma control [35,36,37,38].

This pathological state was accompanied by a profound disruption of immune homeostasis. We observed significant upregulation of Th2-associated cytokines (IL-4, IL-13, IL-25, IL-33) and a Th2-skewed response (Figure 5), alongside elevated IgE, particularly under co-exposure conditions. These patterns are consistent with experimental and epidemiological data showing that PM_2.5_ can damage the airway epithelial barrier, induce oxidative stress, and promote Th2 dominance, thereby enhancing IgE production and eosinophilic inflammation [39,40,41,42,43]. From a humidity perspective, sustained indoor RH above ~60% is associated with dampness-related microbial growth, allergen amplification, and increased asthma symptoms [6,44,45,46]. Our results suggest that high RH may facilitate particle–mucus interactions and antigen presentation, thereby intensifying the Type I hypersensitivity triggered by PM2.5 [5,12,13,47].

### 4.2. The TRPV4–MAPK Axis: The Core Mechanism

A central contribution of this study is the identification of TRPV4–MAPK signaling as a key transduction pathway linking environmental co-exposure to inflammatory amplification. TRPV4 is a polymodal cation channel that responds to osmotic, mechanical, and thermal stimuli and is abundantly expressed in airway epithelial and alveolar cells [48]. Previous work has shown that TRPV4 activation can increase intracellular Ca^2+^, modulate epithelial barrier function, and promote pro-inflammatory signaling [17].

In our model, co-exposure to PM_2.5_ and 90% RH in OVA-sensitized mice was associated with robust TRPV4 activation and subsequent phosphorylation of p38 and JNK, whereas ERK1/2 levels remained largely unaffected (Figure 6 and Figure 7). This clear divergence indicates that p38 and JNK are the predominant downstream mediators of TRPV4-dependent stress in this model. This pattern is compatible with the concept of “hierarchical oxidative stress”, in which stress-activated kinases (e.g., p38 and JNK) play dominant roles in propagating environmentally driven inflammatory responses [23,49,50,51]. p38 MAPK has been implicated in the production of TNF-α, IL-8, and other pro-inflammatory mediators, whilst JNK contributes to immune cell recruitment and smooth muscle hyperresponsiveness in asthma [20,21,23,50]. The relatively modest involvement of ERK1/2 suggests that PM_2.5_ + high RH predominantly engages stress- and inflammation-related MAPK branches rather than proliferative pathways. Importantly, pharmacological inhibition of TRPV4 with HC-067047 significantly reduced p38/JNK phosphorylation and attenuated downstream cytokine production, supporting a regulatory role for TRPV4 upstream of MAPK activation in this co-exposure context [17,18].

### 4.3. Oxidative Stress and NF-κB Regulation

Beyond sensory and kinase signaling, oxidative stress is a major driver of tissue damage and chronic airway remodeling. PM_2.5_ can disturb intracellular calcium homeostasis, promote excessive ROS generation, and deplete antioxidant defenses, thereby triggering inflammatory cascades [52]. In the present study, co-exposure to PM_2.5_ and 90% RH resulted in elevated NF-κB and AP-1 levels alongside suppression of Nrf2, consistent with a shift towards a pro-oxidant, pro-inflammatory state (Figure 8). This is in line with prior evidence that particulate matter exerts its adverse health effects via hierarchical oxidative stress, with Nrf2 and NF-κB acting as opposing regulators of antioxidant and inflammatory responses [49,53].

Nrf2 serves as the master regulator of cellular antioxidant defense, transcriptionally activating cytoprotective genes including glutathione S-transferases, NQO1, and HO-1 [54]. The observed Nrf2 suppression might be attributed to ROS-mediated Keap1 modification [55], enhanced proteasomal degradation, or competitive NF-κB inhibition [56]. Recent evidence indicates that PM2.5 can suppress Nrf2 nuclear translocation via MAPK-mediated phosphorylation [57], consistent with our concurrent MAPK activation and Nrf2 suppression. This may perpetuate a vicious cycle wherein diminished antioxidant defenses amplify oxidative stress. Importantly, TRPV4 inhibition partially restored Nrf2 levels (Figure 8C), suggesting that TRPV4-mediated calcium influx contributes to Nrf2 suppression.

NF-κB is a central transcription factor that orchestrates the expression of pro-inflammatory cytokines (including IL-13) and adhesion molecules, contributing to airway inflammation and remodeling [58,59]. Our data show that TRPV4 blockade not only attenuated MAPK phosphorylation but also reduced NF-κB activation and IL-13 expression, suggesting that TRPV4-mediated Ca^2+^ influx and ROS generation lie upstream of NF-κB in this model [17,18]. Taken together, these findings indicate that TRPV4–MAPK–NF-κB signaling forms a tightly coupled axis linking humidified PM_2.5_ exposure to oxidative stress, Th2 cytokine production, and structural airway changes.

Despite the insights provided by this study, several limitations warrant consideration. First, while intranasal instillation was employed to ensure precise dosing [8], future studies could employ whole-body inhalation chambers to further simulate the long-term dynamics of ambient exposure under fluctuating humidity. Second, as the toxicity of PM_2.5_ may vary depending on its specific constituents (such as transition metals or PAHs) [60], it would be valuable for future research to integrate detailed chemical characterization to identify which components most significantly contribute to the synergistic effects observed here. Finally, while pharmacological blockade with HC-067047 provided important evidence for the involvement of TRPV4, the application of genetic approaches, such as TRPV4 knockout models or targeted gene silencing, would be a promising direction to further validate these signaling pathways in diverse experimental settings.

## 5. Conclusions

This study demonstrates that high humidity (90% RH) amplifies the pathological effects of PM_2.5_ exposure in OVA-sensitized mice, synergistically exacerbating airway hyperresponsiveness and inflammation. High-dose PM_2.5_ (75 $\mu g/m^{3}$) + 90% RH was associated with marked allergic asthma aggravation, occurring concurrently with robust TRPV4-MAPK pathway activation, whereas low-dose PM_2.5_ (35 $\mu g/m^{3}$) + 90% RH elicited a weaker response, suggesting dose-dependent modulation of asthma severity. Pharmacological inhibition of TRPV4 using HC-067047 significantly alleviated pathological outcomes, supporting a mechanistic role for this pathway. Further analysis revealed selective activation of stress-responsive MAPK branches (p38 MAPK and JNK) with minimal ERK1/2 involvement, indicating pathway-specific responses to environmental co-exposure.

## Figures and Tables

**Table 1:** The analysis of PM_2.5_ sample composition.

Chemical Components	Weight Percentage	Chemical Components	Weight Percentage
$SiO_{2}$	69–77	CaO	2.5–5.5
${Al}_{2}O_{2}$	8–14	MgO	1.0–2.0
${Fe}_{2}O_{3}$	4–7	$TiO_{2}$	0–1.0
${Na}_{2}O$	1–4	$K_{2}O$	2.0–5.0

Note: Loss on ignition of 2–5%.

**Table 2:** Effects of PM_2.5_ and/or high relative humidity on inflammatory cell recruitment.

Exposure Groups	Eosinophil/Total Cell Percentage	HC-067047 Blocking Groups	Eosinophil/Total Cell Percentage
Saline	1.88% ± 0.02 *	Saline + HC	1.48% ± 0.01
OVA	26.82% ± 0.21 *	OVA + HC	6.85% ± 0.03 #†
OVA + 0.15PM	52.75% ± 0.22 *	OVA + 0.15PM + HC	4.32% ± 0.01 ##††
OVA + 90%RH	40.08% ± 0.16 *	OVA + 90%RH + HC	9.43% ± 0.06 ††
OVA + 0.15PM + 90%RH	71.78% ± 0.23 *#	OVA + 0.15PM + 90%RH + HC	11.23% ± 0.05 ††
OVA + 0.07PM + 90%RH	41.56% ± 0.15 *	OVA + 0.07PM + 90%RH + HC	6.95% ± 0.05 ††

* *p* < 0.05: a significant difference compared with the saline group. # *p* < 0.05, ## *p* < 0.01: different exposure groups compared with the OVA group. † *p* < 0.05, †† *p* < 0.01: the blocking groups compared with the corresponding exposure groups (*n* = 5).

## Data Availability

The datasets generated and/or analyzed during the current study are available from the corresponding author on reasonable request.
